# Mechanism and Reaction Pathways for Microcystin-LR Degradation through UV/H_2_O_2_ Treatment

**DOI:** 10.1371/journal.pone.0156236

**Published:** 2016-06-09

**Authors:** Yafeng Liu, Jing Ren, Xiangrong Wang, Zhengqiu Fan

**Affiliations:** Department of environmental science & engineering, Fudan University, Shanghai, 200433, China; CEA-Saclay, FRANCE

## Abstract

Microcystin-LR (MCLR) is the most common cyanotoxin in contaminated aquatic systems. MCLR inhibits protein phosphatases 1 and 2A, leading to liver damage and tumor formation. MCLR is relatively stable owing to its cyclic structures. The combined UV/H_2_O_2_ technology can degrade MCLR efficiently. The second-order rate constant of the reaction between MCLR and hydroxyl radical (·OH) is 2.79(±0.23)×10^10^ M^−1^ s^−1^ based on the competition kinetics model using nitrobenzene as reference compound. The probable degradation pathway was analyzed through liquid chromatography mass spectrometry. Results suggested that the major destruction pathways of MCLR were initiated by ·OH attack on the benzene ring and diene of the Adda side chain. The corresponding aldehyde or ketone peptide residues were formed through further oxidation. Another minor destruction pathway involved ·OH attack on the methoxy group of the Adda side chain, followed by complete removal of the methoxy group. The combined UV/H_2_O_2_ system is a promising technology for MCLR removal in contaminated aquatic systems.

## Introduction

Cyanotoxins are drinking water contaminants released by harmful strains of cyanobacteria, such as *Microcystis*, *Anabaena*, *Nostoc*, and *Oscillatoria* [1]. Microcystins (MCs), which contain a large family of cyclic heptapeptides, are one of the most toxic cyanotoxin species. The general structure of MCs is cyclo [-D-Ala-L-X-D-MeAsp-L-Z-Adda-D-Glu-Mdha-], in which X and Z are variable L amino acids. Over 90 MC variants have been identified, and microcystin-LR (MCLR) is the most common species found in contaminated aquatic systems [2]. Studies have shown that MCs inhibit protein phosphatases 1 and 2A, leading to liver damage and tumor formation [3]. In addition, sublethal doses of MCs in drinking water are considered one of the key factors in the unusually high occurrence of primary liver cancer [4, 5].

Owing to their cyclic structures, MCs are relatively stable under a wide range of pH values and temperatures. Many of the bodies of water worldwide from which drinking water is obtained exhibit cyanobacterial blooms, and consumption of cyanobacteria-contaminated drinking water is the main cause of exposure to MCs.

A variety of conventional water treatment technologies, including coagulation/sedimentation [6], activated carbon adsorption [7], and membrane separation [8], have been employed to remove cyanotoxins; however, these techniques are ineffective owing to the stable structure of cyanotoxins, so they can’t eliminate or reduce the toxicity as the cyan toxins can’t be removed from the environment thoroughly. Advanced oxidation processes (AOPs) have recently become an effective alternative for remediation or removal of MCs. AOPs typically involve production of hydroxyl radicals (·OH) as oxidizing species, which degrade target organic materials [9]. The rate constants are typically in the order of 10^8^–10^10^ L mol^−1^ s^−1^. Studies have shown that MCs in aqueous solutions are efficiently degraded by TiO_2_/UV [10–12], Fenton and photo-Fenton [13, 14], and ultrasonic irradiation [15, 16] and that ·OH is mainly responsible for MC degradation. The reaction mechanisms and pathways involved in TiO_2_/UV and ultrasonic irradiation have already been proposed [16–18]. Moreover, toxicity experiments demonstrated that degradation of intermediates and byproducts significantly eliminates toxicity of cyanotoxins [10, 12].

Combined UV/H_2_O_2_ technology efficiently degrades MCs [19, 20]. ·OH and UV direct photolysis are both primarily responsible for MCLR degradation in combined UV/hydrogen peroxide (H_2_O_2_) process. However, the reaction mechanism and reaction pathway of MCLR degradation in the UV/H_2_O_2_ process has never been well understood. Previous studies on UV/H_2_O_2_ treatment have shown that direct photolysis of target compounds is assumed to be negligible [21, 22]. However, earlier studies have shown [19] that MCLR degradation is obviously affected by direct photolysis and thus should be taken into consideration [23]. This paper aimed to determine the second-order rate constant of the reaction between MCLR and ·OH and to investigate the reaction pathways involved in the UV/H_2_O_2_ process.

## Materials and Methods

### Chemicals

This study adopted the chemicals used by Changlong Wu [23]. Standard MCLR was purchased from Sigma-Aldrich Co., Ltd. and HPLC-grade acetonitrile (ACN) was purchased from Tedia Co., (USA). H_2_O_2_ (30%, w/w), sodium sulfite, sodium hydroxide, and sulfuric acid were purchased from Shanghai Chemical Reagent Co., Ltd. (Shanghai, China). All reagents are of analytical grade. Ultrapure water was obtained using Milli-Q Water System (Millipore, Bedford, MA, USA).

### Photochemical experiments

Photochemical experiments were performed according to the method described by Changlong Wu [23]. Commercial low-pressure Hg UV lamp (6 W, 254 nm) was used for illumination [S1]. The UV light intensity irradiating the surface of the sample solution was controlled by adjusting the distance between the UV lamp and the quartz bottle and was measured using a luxometer. All experiments were conducted at 25 ± 1°C under atmospheric pressure and UV intensity of 134 μW/cm^2^ at 10 cm. The reaction began by introducing a predetermined dosage of H_2_O_2_ into 3 mL of sample solution in a quartz bottle and by turning on the UV lamp. The exposure time is 10 minutes. The pH of the solution was 5.8 in all experiments. We used 3 mL of 100 mg/L MCLR solution to identify the reaction intermediates.

### Analysis

The method used in the analysis was previously described by Weihua Song [16]. MCLR was analyzed using an HPLC (Agilent 1200) with photodiode array detection under the following conditions: the column used was ZORBAX SB-C18 column (5 μm, 4.6 mm×250 mm, Agilent, USA) and the mobile phase consisted of 40% ACN and 60% water, both containing 0.05% trifluoroacetic acid (TFA). The liquid chromatography-mass spectrometry (LC-MS) system used in this study consisted of a Dionex Ultimate 3000 HPLC Pump, a Dionex Ultimate 3000 auto-sampler, and a Bruker micrOTOF II Mass Spectrometer with an electrospray ionization source. The HPLC column used was ZORBAX SB-C18 column (5 μm, 4.6 mm×250 mm, Agilent, USA). Moreover, the injection volume of the treated sample was 80 μL, and the mobile phase used was water and ACN, both containing 0.05% TFA. Gradient elution was performed based on the method described by Liu *et al*. [18]. Mass spectral data were also obtained in the positive ion mode by full scanning from m/z 400 to 1200.

### Rate Constant Calculation

Competition kinetics experiments were conducted to evaluate the rate constant (*k*_OH_) between ·OH and MCLR. Nitrobenzene (NB) [*k*_OH_ = 4.0×10^9^ M^−1^ s^−1^] was chosen as reference compound because NB is difficult to directly photodegrade and is easily analyzed by HPLC. Moreover, NB has been successfully applied in similar experiments [23, 24]. Direct photolysis can be expressed by Eq 1 [25].

$$-\frac{\text{d}[\text{MCLR}]}{\text{d}t}=k_{\text{d}}[\text{MCLR}]=k_{\text{S},\;\text{MCLR}}\Phi_{\text{MCLR}}[\text{MCLR}]$$ (1)

where *k*_d_ is the pseudo-first-order reaction rate constant; *k*_S_, _MCLR_ is the rate constant of first order reaction by MCLR (E mol^−1^s^−1^); and Φ_MCLR_ is the quantum of MCLR (percentage of excited stage photons).

Assuming that direct photolysis of NB is negligible, destruction rates of MCLR and NB can be expressed by Eqs 2 and 3, respectively.

$$r_{\text{MCLR}}=k_{\text{OH},\;\text{MCLR}}{\left[\cdot \text{OH}\right]}_{\text{SS}}+k_{\text{S},\;\text{MCLR}}\Phi_{\text{MCLR}}f_{\text{MCLR}}$$ (2)

$$r_{\text{NB}}=k_{\text{OH},\;\text{NB}}{\left[\cdot \text{OH}\right]}_{\text{SS}}$$ (3)

where [·OH] SS is the steady-state concentration of ·OH. The fraction of UV absorbed by MCLR (*f*_MCLR_) is determined by Eq 4.

$$f_{\text{MCLR}}=\frac{\varepsilon_{\text{MCLR}}[\text{MCLR}]}{\varepsilon_{H_{2}O_{2}}[{\text{H}}_{2}{\text{O}}_{2}]+\varepsilon_{\text{NB}}[\text{NB}]+\varepsilon_{\text{MCLR}}[\text{MCLR}]}$$ (4)

*k*_OH_ of MCLR can be calculated by Eq 5.

$$k_{\text{OH},\;\text{MCLR}}=k_{\text{OH},\;\text{NB}}\frac{r_{\text{MCLR}}-k_{\text{d}}f_{\text{MCLR}}}{r_{\text{NB}}}$$ (5)

## Results and Discussion

### ·OH rate constant

In order to calculate the·OH rate constant, we use Nitrobenzene(NB) concentrations from 10 μM to 100 μM were added into MCLR working solution (5 μM), and the solutions were treated with UV/H_2_O_2_ process by using 500 μM of H_2_O_2_. To determine the reaction rate constant of direct photolysis, we treated 5 μM of NB-free MCLR working solutions with UV irradiation alone under the same conditions as that in UV/H_2_O_2_ treatment, including the same UV intensity, pH value, and temperature (Table 1). *k*_OH_ of MCLR was constant under various concentrations, and the average value was 2.79 (±0.23)×10^10^ M^−1^ s^−1^.

**Table 1:** Parameters determined under different nitrobenzene concentrations.

*C*_*NB*_*(μM)*	*C*_*MCLR*_*(μM)*	*r*_*NB*_ *(M*^*-1*^*s*^*-1*^*)*	*r*_*MCLR*_ *(M*^*-1*^*s*^*-1*^*)*	*f*_*MCLR*_	*k*_*d*_ *(M*^*-1*^*s*^*-1*^*)*	*k*_OH,MCLR_ (M^-1^s^-1^)(×10^10^)
10.0	5.0	0.0638	0.4572	0.310	0.0362 (R^2^ = 0.9124)	2.80
19.5	4.4	0.0525	0.3318	0.176	0.0375 (R^2^ = 0.8925)	2.48
50.0	5.0	0.0544	0.3521	0.088	0.0362 (R^2^ = 0.9124)	2.57
88.5	3.7	0.0292	0.2216	0.039	0.0401 (R^2^ = 0.8792)	3.01
87.1	4.9	0.0416	0.3171	0.052	0.0368 (R^2^ = 0.9237)	3.03
100.0	5.0	0.0292	0.2105	0.046	0.0362 (R^2^ = 0.9124)	2.86

In a detailed assessment of the reactivity of ozone and ·OH toward different cyanotoxins, a bimolecular rate constant of 1.0×10^10^ M^−1^s^−1^ was previously determined by the reaction of ·OH with MCLR [26]. Competitive kinetics with pulsed radiolysis and transient absorption measurements were employed to determine the reaction rate of ·OH with MCLR, and the reaction rate is 2.3 (±0.1)×10^10^ M^−1^s^−1^ [27]. The difference between these measured rate constants is possibly caused by variations in experimental conditions and in the methods of determination [27]. Moreover, the pH of the solution influences conformation, charge, or both and can exert pronounced effect on the reactivity of MCLR. The rate constant of 1.0×10^10^ M^−1^s^−1^ was also determined under phosphate-buffered conditions at pH 7 [26], whereas the rate constants were determined under natural pH both in this paper and in Song’s study [27]. Hence, the results determined in this paper are closer to that reported by Song *et al*. [27] compared with that reported by Onstad *et al*. [26].

### LC-MS identification of byproducts of MCLR subjected under combined UV/H_2_O_2_ treatment

To separate and detect the degradation products, considerably higher concentration of MCLR agent was employed for the degradation experiment compared with that found in the environment. The intermediates were characterized based on the MS peak area criteria: the peaks must have a signal-to-noise ratio of 3, and the peak areas of treated sample must be at least two times larger than that if the initial sample, if the peak was found in the initial sample [17]. The LC-MS analysis under various reaction times revealed 9 decomposition products at detectable levels (Table 2). Moreover, the structural assignments of the degradation products of MCLR from the combined UV/H_2_O_2_ treatment were based on the analysis of the total ion chromatogram (TIC) and on the corresponding mass spectra. The masses of the different products were determined from the (M+H)^+^m/z peaks corresponding to the molecular ion [16].

**Table 2:** Reaction intermediates of MCLR degraded by the combined UV/H_2_O_2_ treatment.

*No*.	*Peak (m / z)*	RT (min)
1	795.4	6.0
2	795.4	6.7
3	835.4	7.1
4	811.4	6.1
5	1029.5	10 ~ 18
6	1011.5	15 ~ 20
7	1045.5	18.2
8	1045.5	20.9
9	1027.5	21.2
10	1009.6	21.4
11	995.5	21.7
12	995.5	22.3
13	965.6	25.2

The typical reaction between ·OH and target organic substance involves three competition pathways: addition, hydrogen abstraction, and electron abstraction. The rates of reaction of ·OH with different reaction sites in MCLR molecular vary significantly.

The product with (M+H)^+^ at m/z 1029.5 was a major product and displayed a large time span in TIC (10 min < t < 18 min). This product corresponds to the addition of 34 mass units into MCLR and can result from addition of two ·OH into the diene in the Adda side chain through hydroxyl addition reaction. The first ·OH was incorporated into the double bond to form allyl radical (RCH = CH-R_2_C·) and then a second ·OH reacted with the allyl carbon center to form the double hydroxylation derivative MCLR. The product ion (M+H)^+^ at m/z 1029.5 during TiO_2_ photocatalytic oxidation (PCO) was reported and assigned as a diol [12,18].

**Fig 1:**
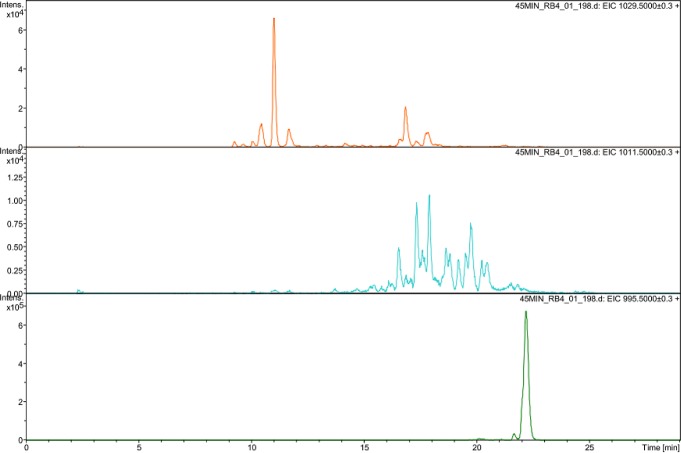
LC-MS chromatograph of MCLR after 45 min of UV/H_2_O_2_ treatment.

More than six peaks with a mass spectrum showing a molecular ion (M+H)^+^ at m/z 1029.5 were observed in the TIC analysis (Fig 1). These products are proposed to be formed by geometrical isomerization of MCLR following UV_254_ irradiation and ·OH attack on the diene, leading to isomeric diols [18]. Under UV_254_ irradiation, the (4E), (6E) of the Adda configuration can be converted into (4E), (6Z) or (4Z), (6E) [28, 29], and this phenomenon was supported by the occurrence of two peaks corresponding to (M+H)^+^ at m/z 995.5 in TIC (Table 2). Geometrical isomerization of MCLR provided precursors for the dihydroxyl-MCLR. In addition, double hydroxylation can occur on any of the double-bond pairs (C4-C5 and C6-C7) and can result in stereoisomeric forms of 1, 2- and 1, 4- dihydroxylated adducts of MCLR [17,27].

Dihydroxy-MCLR was further oxidized with cleavage of the dihydroxylated bond, resulting in different cleavage products depending on the location of the dihydroxy substitutes in the precursors. The (M+H)^+^ ion at m/z 795.4 in the UV/H_2_O_2_ process is probably a cleavage products at positions C4-C5 on the Adda side chain, and the (M+H)^+^ ion at m/z 835.4 is the cleavage products at positions C6-C7 on the Adda side chain. Moreover, the (M+H)^+^ ion at m/z 811.3 is consistent with a carboxylic acid structure formed by bond cleavage of (M+H)^+^ ion at m/z 795.4.

The (M+H)^+^ ion at m/z 1011.5 intermediate was reported in other AOTs, such as ultrasonic irradiation [16] and PCOs. This ion corresponded to the addition of 16 mass units to MCLR and can result from addition of one ·OH into the diene or the aromatic ring in the Adda side chain through hydroxyl substitution reaction.

In the case of diene substitution reaction, an OH group substituted the hydrogen of C7 in the Adda side chain and formed an enol MCLR. The enol MCLR rapidly isomerized into a more stable tautomer of ketone MCLR, which then underwent a series of oxidation-induced bond cleavage reactions to form a ketone-derived ((M+H)^+^ ion at m/z 835.4) and aldehyde-derived ((M+H)^+^ ion at m/z 795.4).

In the case of aromatic substitution, the aromatic ring in the Adda side chain was attacked by ·OH, and the probable reaction mechanism involved the addition of one ·OH into one of the aromatic double bonds, forming a carbon-centered radical; the reaction of the carbon-centered radical with oxygen formed a peroxy radical, and finally, the elimination of hydroperoxyl radical yielded a phenolic derivative. In this case, *ortho*, *para*, or *meta* substitution usually occurs [16, 27]. The first aromatic hydroxylation increases the electron density of the aromatic ring and thus eletrophilic reactions (such as ·OH attack) proceed faster [16, 17, 30]. Therefore, the first hydroxylation of the aromatic ring was followed by a second one as demonstrated by detection of (M+H)^+^ ion at m/z 1027.5, consistent with the result of addition of a second hydroxyl group to the phenyl group.

The reaction rates of the ·OH with different reaction sites present in MCLR are expected to vary significantly. The reaction rate of ·OH addition to the aromatic ring is the fastest among the competing processes because this process generally occurs at a nearly diffusion-controlled rate [16, 31]. This finding is also supported by the present study given that the m/z 1011.5 ion occurred at 2 min, whereas the other product ions did not occur until 5 min. Oxidation of both the conjugated diene and phenyl ring resulted in a product with (M+H)^+^ ion at m/z 1045.5. This product was not observed in previous PCOs [18] but was observed in ultrasonic treatment [16, 32]. This product can undergo further oxidation of diol or cleavage of the diene, yielding a cleavage product (the (M+H)^+^ ion at m/z 835.4) [16].

In addition, the reaction pathway involving the removal of methoxy group of the Adda chain has been observed in the analogous products during the UV/H_2_O_2_ process; this pathway was also proposed during PCO of MCLR [17]. The (M+H)^+^ ion at m/z 1009.5 ion was assigned to the formic acid ester MCLR derivative, which was followed by complete removal of the methoxy group of Adda chain, thereby forming the (M+H)^+^ ion at m/z 965.5 ion, DmADDA. These molecular ions are rarely observed in other AOT processes.

**Fig 2:**
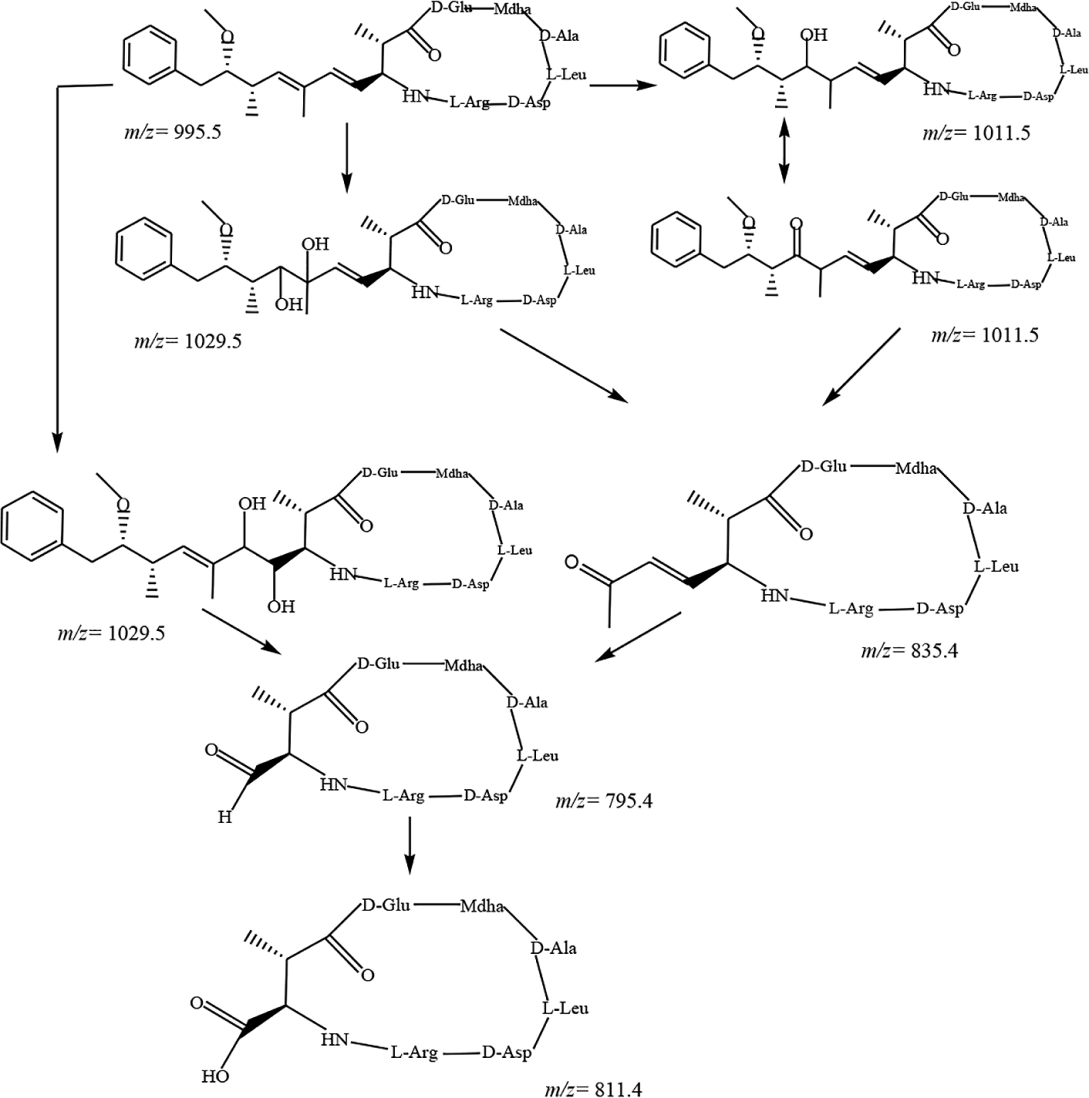
Byproducts and proposed reaction pathways for the combined UV/H_2_O_2_ degradation of the conjugated carbon double bonds of Adda side chain of MCLR.

**Fig 3:**
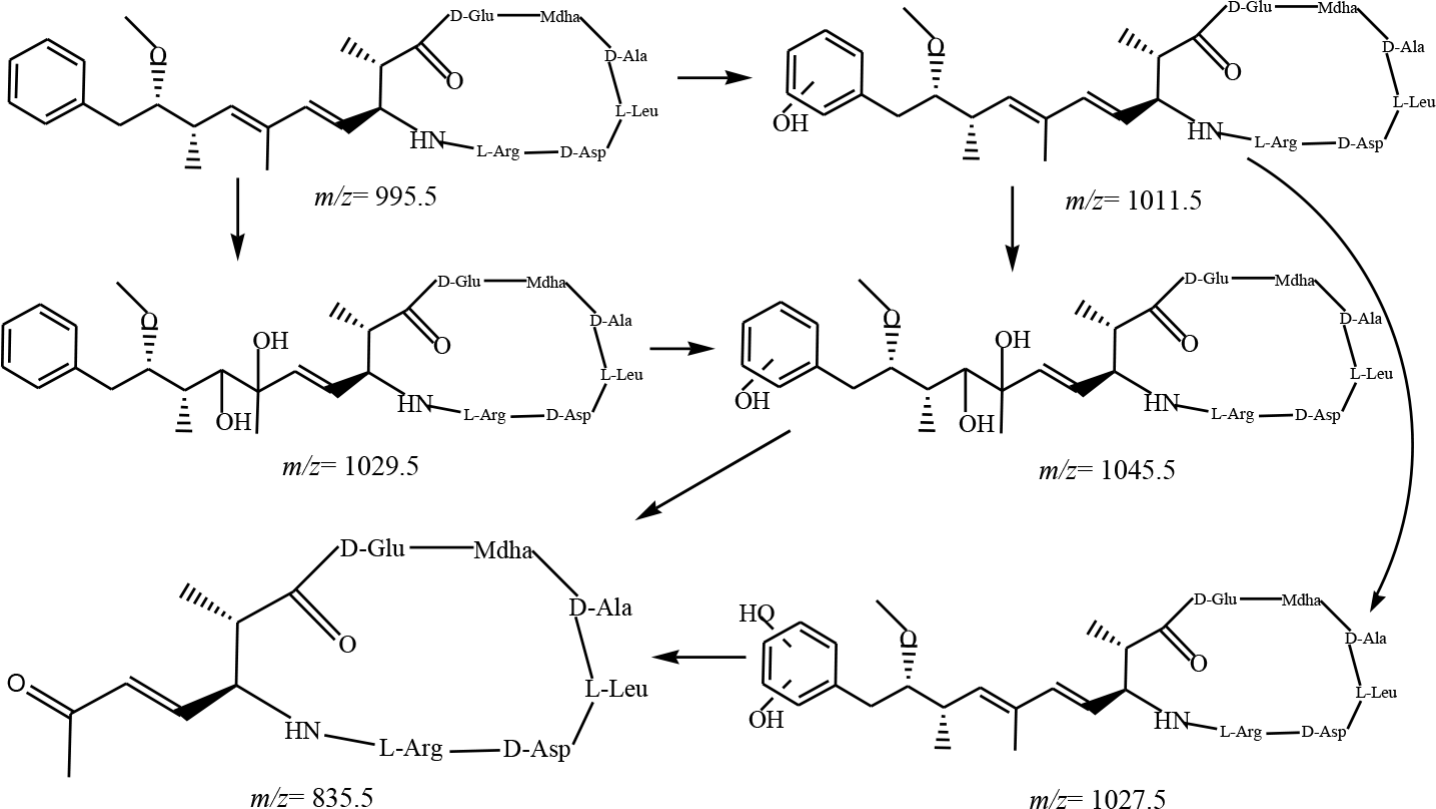
Byproducts and proposed reaction pathways for the combined UV/H2O2 degradation of the aromatic ring of Adda of MCLR.

**Fig 4:**
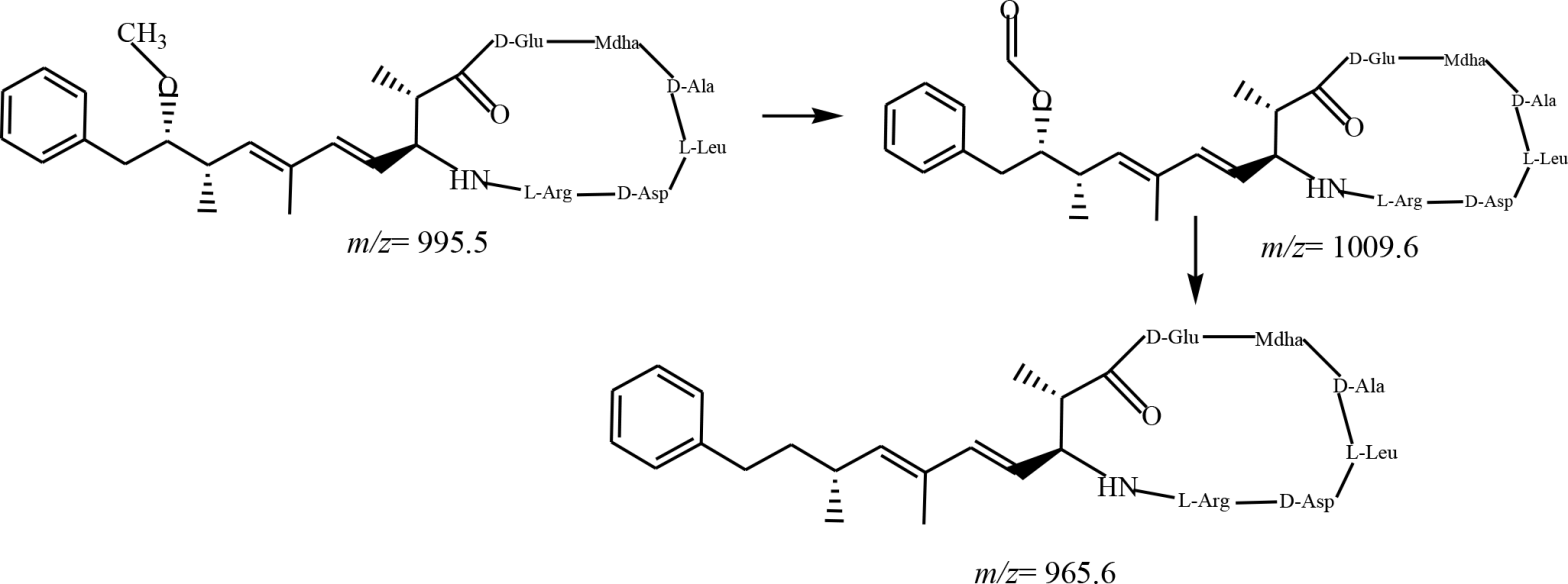
Byproducts and proposed reaction pathways for the combined UV/H2O2 degradation of the methoxy group of Adda of MCLR.

A summary of the proposed reaction pathways involved in the UV/H_2_O_2_ process are illustrated in Figs 2–4.

**Fig 5:**
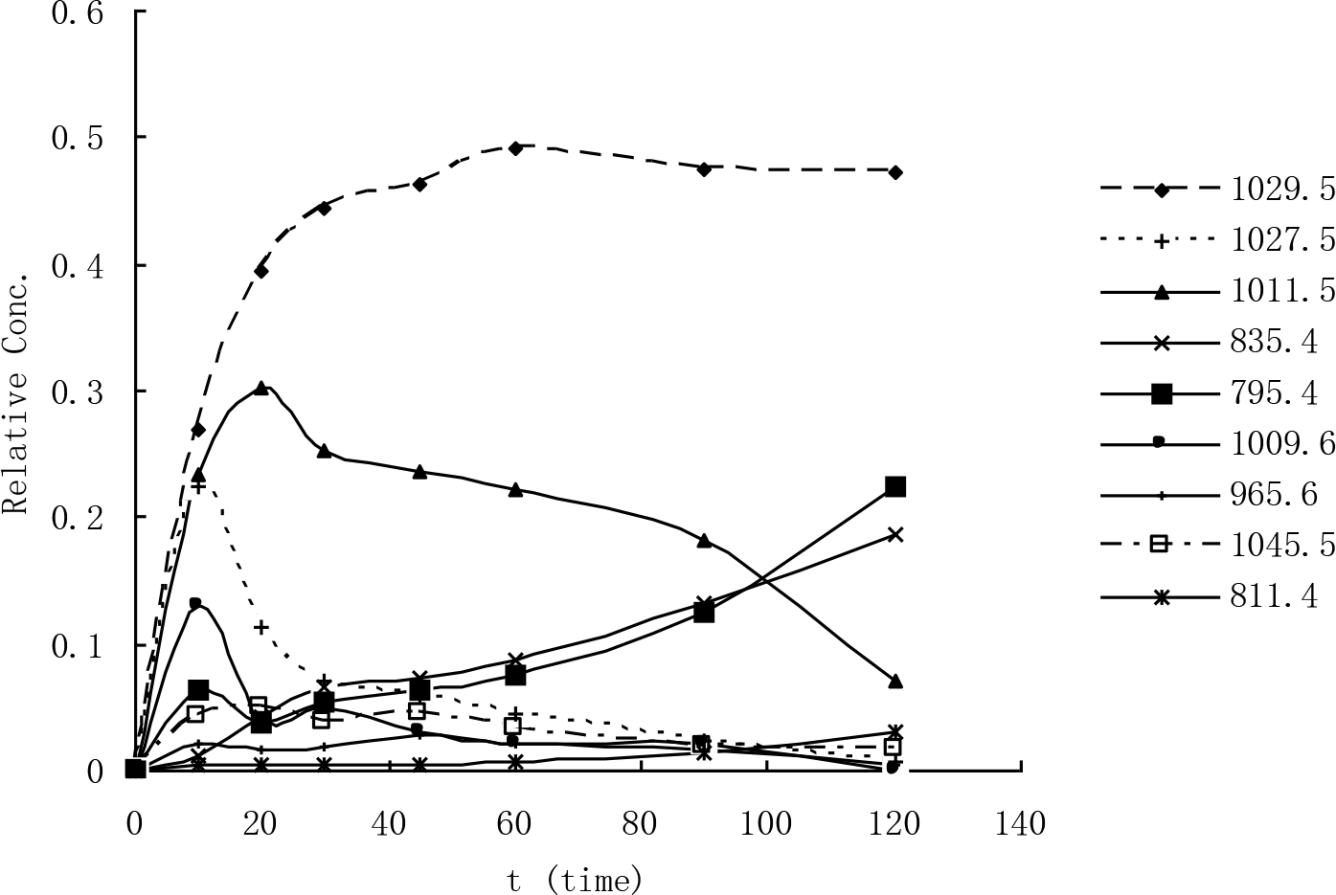
Relative yield of product ions versus time in the UV/H2O2 treatment.

The relative concentration of the reaction products of MCLR was monitored using LC-MS as a function of treatment time (Fig 5). Given that the products exhibit similar structures, their response factors (peak intensity/molecule) were assumed to be similar, and their peak intensities were taken as indicator of their relative yields. The early appearance of the peaks was mainly assigned to the phenolic and diol products, and the reaction pathways involved accounted for over 90% of the total reactions.

### Toxicity analysis of the degradation intermediates

Degradation of MCLR through the combined UV/H_2_O_2_ treatment was primarily caused by ·OH attack and direct photolysis. The main intermediates of MCLR decomposition through the combined UV/H_2_O_2_ process can be classified into two structural groups: group 1, consisting of ring peptides containing an Adda moiety ((M+H)^+^ ion at m/z 1029.5, 1011.5, 1045.5, 1027.5, 1009.5, and 965.5); and group 2, consisting of ring peptides without an Adda moiety ((M+H)^+^ ion at m/z 835.4, 795.4, and 811.3). Given that the conjugated diene structure of Adda in MCLR is essential for inhibition of protein phosphatases 1 and 2A [33, 34], the products of peptide residues without an Adda moiety are expected to be detoxified. Moreover, the other products, which underwent modification (e.g., dihydroxylation) in Adda side chain, also eliminate toxicity [33–35]. Therefore, the combination of UV/H_2_O_2_ is a promising technology for MC removal in bodies of water contaminated with cyanobacteria.

## Conclusions

The combined UV/H_2_O_2_ system can effectively degrade MCLR in aqueous solution. Using nitrobenzene (NB) as competition reference compound, we developed a competition kinetic model for degradation of MCLR by UV/H_2_O_2_ by employing the pseudo-first-order equation and steady-state approximation. Using the competition kinetic model, we determined the second-order rate constant of the reaction between MCLR and ·OH to be 2.8 (±0.21)×10^10^ (M^−1^ s^−1^).

MCLR degradation through UV/H_2_O_2_ treatment mainly involved ·OH attack, oxidation, and UV_254_ direct photolysis. The main sites of MCLR molecule attacked by ·OH were the conjugated diene bond, benzene ring, and methoxy group of the Adda side chain.

Based on the molecular weight of the products and the reaction mechanism between ·OH and peptides or protein, three main degradation pathways were proposed as follows: 1) ·OH attacked the conjugated diene bond of Adda side chain through electrophilic addition reaction and produced dihydroxylated-MCLR, and then the hydroxylated C4-C5 or C6-C7 bond of Adda was cleaved through further oxidation to form aldehyde or ketone peptide residues, which were subsequently oxidized into their corresponding carboxylic acids. 2) ·OH attacked the benzene ring and formed benzene hydroxylation and/or benzene dihydroxylation through electrophilic substitution reaction followed by further oxidation to form aldehyde or ketone peptide residues. 3) ·OH attacked the methoxy group of the Adda side chain through hydrogen abstraction reaction to form formic acid-(MCLR), leading to the complete removal of the methoxy group. Pathways 1 and 2 probably accounted for over 90% of the total degradation reactions.

## Supporting Information

S1 Fig The UV254 exposure apparatus for MCLR degradation by UV/H2O2 treatment. (DOC)
