# Supplementary material for: Mechanism and Reaction Pathways for Microcystin-LR Degradation through UV/H2O2 Treatment
Source: PLoS One. 2016 Jun 9;11(6):e0156236. doi: 10.1371/journal.pone.0156236 (PMC4900665; doi:10.1371/journal.pone.0156236)
Supplement: S1 Fig — (DOC) [file pone.0156236.s001.doc]

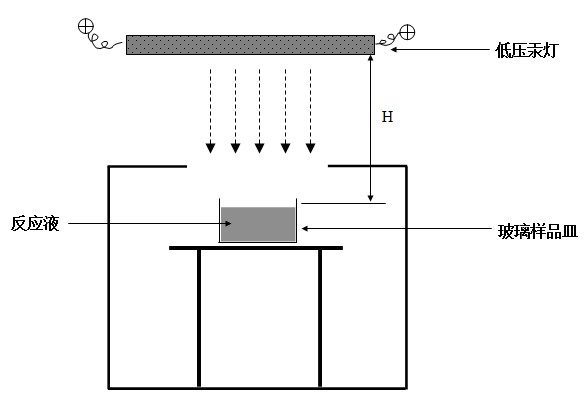


Figure1 The UV_254_ exposure apparatus for MCLR degradation by UV/H_2_O_2_ treatment

a

c

b

d

a

b

c

d

a

A

B

C

Figure2. HPLC chromatograms of MCLR degradation by UV_254_、UV/H_2_O_2_ and H_2_O_2_ treatment (A, UV_254_ treatment ; B, UV/ H_2_O_2_ treatment; C, H_2_O_2_ treatment)

UV/ H_2_O_2_

UV

H_2_O_2_

Figure 3 Remaining MCLR/initial MCLR (C/C_0_) versus time in the UV_254_, UV/H_2_O_2_ and H_2_O_2_ treatment

Figure 4 Remaining MCLR/initial MCLR (C/C_0_) versus time as a function of initial H_2_O_2_ concentration, C_0_=14.5 mg/L; pH 8.0; T=25±1℃

Figure 5 Remaining MCLR/initial MCLR (C/C_0_) versus time as a function of initial MCLR concentration, pH 8.0, T=25±1℃.

Figure 6 Remaining MCLR/initial MCLR (C/C_0_) versus time as a function of initial pH, T=25±1℃

Figure 7 Remaining MCLR/initial MCLR (C/C_0_) versus time as a function of initial UV intensity, C_0_=14 mg/L; pH 8.0; T=25±1℃


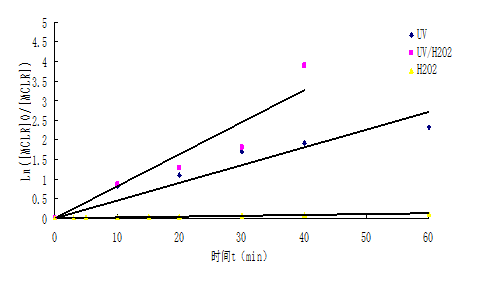


Figure 8 Fitted pseudo-first-order kinetic curve of MCLR degradation by UV_254_、H_2_O_2_ and UV/H_2_O_2_ treatment

Table 1 Fitted pseudo-first-order kinetic parameters of MCLR degradation with different processes

| system | Rate equation | *k*, min^-1^ | *t*_1/2_ , min | *R*^2^ |
| --- | --- | --- | --- | --- |
| UV/H_2_O_2_ | Ln(C_0_/C)=0.082*t* | 0.082 | 8.45 | 0.89 |
| UV | Ln(C_0_/C)=0.045*t* | 0.045 | 15.40 | 0.87 |
| H_2_O_2_ | Ln(C_0_/C)=0.0021*t* | 0.0021 | 330.07 | 0.94 |

Figure 9 Fitted pseudo-first-order kinetic curve of MCLR degradation with different UV_254_ intensity

Table 2 Fitted pseudo-first-order kinetic parameters of MCLR degradation with different initial MCLR concentration

| MCLR（mg/L） | *k*, min^-1^ | *t*_1/2_ , min | *R*^2^ |
| --- | --- | --- | --- |
| 8.5 | 0.1485 | 4.7 | 0.7349 |
| 9.6 | 0.1068 | 6.5 | 0.7708 |
| 14.8 | 0.0854 | 8.1 | 0.7878 |
| 23 | 0.0519 | 13.4 | 0.8855 |

Table 3 Fitted pseudo-first-order kinetic parameters of MCLR degradation with different pH

| MCLR（mg/L） | pH | *k*, min^-1^ | *t*_1/2_ , min | *R*^2^ |
| --- | --- | --- | --- | --- |
| 14.6 | 4.3 | 0.1294 | 5.4 | 0.9725 |
| 15.5 | 5.6 | 0.1533 | 4.5 | 0.9317 |
| 14.7 | 7.2 | 0.1801 | 3.8 | 0.8861 |
| 14.8 | 8.4 | 0.0753 | 9.2 | 0.9201 |
| 15.0 | 9.8 | 0.0631 | 11.0 | 0.9373 |

Figure 10 HPLC analysis of MCLR and NB degradation by UV/H_2_O_2_ treatment versus time（T=0 min、5 min、10 min、15 min）.(A, λ=262.4 nm; B, λ=238.4 nm)

A

B

Table 4 Parameters determined under different concentration of nitrobenzene

| C_NB_ (µM) | C_MCLR_ (µM) | *r*_NB_ (min^-1^) | *r*_MCLR_ (min^-1^) | f_MCLR_ | *k*_d_ (min^-1^) | *k*_OH,MCLR_ (M^-1^s^-1^)  (×10^10^) |
| --- | --- | --- | --- | --- | --- | --- |
| 10.0 | 5.0 | 0.0638 | 0.4572 | 0.310 | 0.031 (*R*^2^=0.9124) | 2.81 |
| 19.5 | 4.4 | 0.0525 | 0.3317 | 0.176 | 0.0292 (*R*^2^=0.8925) | 2.49 |
| 50.0 | 5.0 | 0.0544 | 0.3521 | 0.088 | 0.031 (*R*^2^=0.9124) | 2.57 |
| 88.5 | 3.7 | 0.0292 | 0.2216 | 0.039 | 0.029 (*R*^2^=0.8792) | 3.02 |
| 87.1 | 4.9 | 0.0416 | 0.3171 | 0.052 | 0.0283 (*R*^2^=0.9237) | 3.03 |
| 100.0 | 5.0 | 0.0292 | 0.2105 | 0.046 | 0.031 (*R*^2^=0.9124) | 2.86 |
